# Intra-cervical lymphatic immunotherapy for dust mite-induced allergic rhinoconjunctivitis in children: a 3-year prospective randomized controlled trial

**DOI:** 10.3389/fimmu.2023.1144813

**Published:** 2023-08-01

**Authors:** Qixing Wang, Kai Wang, Yang Qin, Weijun Huang, Yin Li, Qingqing Yu, Yu Xiong, Yingwei Guo, Rui Zheng, Jun Tang

**Affiliations:** ^1^ Zhuhai Campus of Zunyi Medical University, Zhuhai, China; ^2^ Department of Otolaryngology, First People’s Hospital of Foshan, Foshan, China; ^3^ The First Clinical College of Guangdong Medical University, Zhanjiang, China; ^4^ Department of Otorhinolaryngology-Head and Neck Surgery, The Third Affiliated Hospital of Sun Yat-sen University, Guangzhou, China; ^5^ Department of Allergy, The Third Affiliated Hospital of Sun Yat-sen University, Guangzhou, China

**Keywords:** allergic rhinoconjunctivitis, intra-cervical lymphatic immunotherapy, subcutaneous immunotherapy, dust mite, children, allergen

## Abstract

**Background:**

Pediatric allergic rhinoconjunctivitis has become a public concern with an increasing incidence year by year. Conventional subcutaneous immunotherapy (SCIT) has long treatment time, high cost and poor compliance. The novel immunotherapy significantly shortens the course of treatment by directly injecting allergens into cervical lymph nodes, which can perform faster clinical benefits to children.

**Objective:**

By comparing with SCIT, this study aimed to evaluate the long-term efficacy and safety of intra-cervical lymphatic immunotherapy (ICLIT).

**Methods:**

This is a prospective randomized controlled study. A total of 50 allergic rhinoconjunctivitis children with dust mite allergy was randomly divided into ICLIT group and SCIT group, receiving three cervical intralymphatic injections of dust mite allergen or three years of subcutaneous injection, separately. Primary outcomes included total nasal symptom scores (TNSS), total ocular symptom scores (TOSS), total symptom scores (TSS), total medication scores (TMS), and total quality of life score. Secondary outcomes included pain perception and adverse reactions during treatment. Other secondary outcome was change in *Dermatophagoides pteronyssinus* (Derp) and *Dermatophagoides farina* (Derf) -specific IgE level.

**Results:**

Both groups had significantly decreased TNSS, TOSS, TSS, TMS, and total quality of life score after 36 months of treatment (p<0.0001). Compared with SCIT, ICLIT could rapidly improve allergic symptoms (p<0.0001). The short-term efficacy was consistent between the two groups (p=0.07), while the long-term efficacy was better in SCIT group (p<0.0001). The pain perception in ICLIT group was lower than that in SCIT group (p<0.0001). ICLIT group was safer. Specifically, the children had only 3 mild local adverse reactions without systemic adverse reactions. The SCIT group had 14 systemic adverse reactions. At last, the serum Derp and Derf-specific IgE levels in ICLIT and SCIT groups decreased 3 years later (p<0.0001).

**Conclusion:**

ICLIT could ameliorate significantly the allergic symptoms in pediatric patients with an advantage in effectiveness and safety, besides an improved life quality including shortened period of treatment, frequency of drug use and pain perception.

**Clinical trial registration:**

https://www.chictr.org.cn/, identifier ChiCTR1800017130.

## 1 Introduction

Allergic rhinoconjunctivitis refers to immunoglobulin E (IgE)-mediated transmitter release triggered locally by inhaled allergens in the nasal mucosa, and type I allergic reactions involving multiple cells and immunoactive factors (1). Allergic rhinoconjunctivitis is a widely prevalent chronic non-infectious inflammatory disease that can cause nasal itching, sneezing and runny nose, and even bronchial asthma in severe cases. Prevalence of allergic rhinoconjunctivitis has increased significantly in recent years, affecting 20%-40% children worldwide, which places a heavy psycho-economic burden on their families (2). Epidemiological studies in China have shown that the prevalence of allergic rhinoconjunctivitis is approximately 10.5%-31.4% in adults (3) and up to 18.10%-49.68% in children (4, 5).

Dust mite-induced allergy accounts for 85% of all patients with respiratory tract allergy, of which *Dermatophagoides pteronyssinus* (Derp) and *Dermatophagoides farinae* (Derf) are two important allergens causing nasal allergy reactions among children, especially those live in southern China (6). Based on the combination of prevention and treatment, the World Health Organization (WHO) recommends a four-in-one scheme for allergic rhinoconjunctivitis (7), whose standardized allergen immunotherapy (AIT) can induce specific tolerance, reduce the risk of emerging allergic diseases, as well as prevent the evolution of asthma in children. Besides, the plan is the only causal treatment that alters the natural course of allergic diseases by modulating immune mechanisms (8).

As a conventional immunotherapy, subcutaneous immunotherapy (SCIT) has been confirmed by several meta-analyses and systematic reviews in terms of its sustained benefit and long-term clinical efficacy on allergic rhinoconjunctivitis children (9, 10). However, around 13%-89% patients have poor compliance due to the disadvantages of SCIT such as long period of treatment and high frequency of injection. In addition, the Coronavirus disease 2019 (COVID-19) brings more inconvenience to hospital for children and their families, thus less than 5% of patients would like to receive AIT (11).

Intra-cervical lymphatic immunotherapy (ICLIT) is a novel immunotherapy for patients with dust mite allergy, and its short-term efficacy and safety have been proved in clinical studies (12). According to color Doppler ultrasound-guided positioning, ICLIT injects standardized allergen extract into cervical lymph nodes at 0, 4 weeks and 8 weeks with a total course of 2 months, which significantly shortens treatment process, reduces incidence of adverse reactions and medical cost. However, no known reports have focused on exploring the long-term efficacy and safety of ICLIT, or comparing it with traditional immunotherapy.

This prospective randomized controlled study aimed to evaluate the long-term efficacy and safety of ICLIT among children with allergic rhinoconjunctivitis.

## 2 Materials and methods

### 2.1 Study population

A total of 50 pediatric patients at outpatient department of otorhinolaryngology were recruited in our hospital from June 2018 to September 2018, and were then randomly divided into ICLIT group and SCIT group with a ratio of 1:1.

The inclusion criteria were as follows: (1) aged 6 to 17 years; (2) confirmed history of dust mite-induced allergic rhinoconjunctivitis according to Allergic Rhinitis and its Impact on Asthma (ARIA); (3) main symptoms included four nasal symptoms (i.e., nasal itching, sneezing, runny nose, and nasal congestion) and two ocular symptoms (i.e., eye itching and lacrimation) (13). The diagnosis of Derp and Derf -induced allergic rhinoconjunctivitis was confirmed by positive results in skin prick test (SPT, its positive reaction grade is expressed by skin index (SI), which is the ratio of the mean allergen wind cluster diameter to the mean histamine wind cluster diameter. +,0.3≤SI<0.5; ++,0.5≤SI<1; +++,1≤SI<2; ++++,SI≥2) and presence of serum dust mite-specific IgE (sIgE≥0.35 kU/L). All participants were able to cooperate and none of them reported allergic asthma. Patients were excluded if (1) they had allergen sensitization other than dust mites; (2) they had asthma, urticaria and other allergic diseases; (3) they were unable to cooperate with the treatment due to serious psychological disorders; (4) they were complicated with severe adenoid or tonsillar hypertrophy affecting sleep; (5) they had severe congenital, immune, and cardiovascular disease; (6) they and/or their families could not understand risks and limitations of the treatment.

### 2.2 Randomization

Eligible patients were randomly allocated in a 1:1 ratio to ICLIT or SCIT group. With random-number tables, we selected 50 random numbers in sequence starting from any random number and put them in numerical order. Numbers 1-25 and 26-50 were divided into ICLIT group and SCIT group, respectively. The randomization was conducted using SPSS, Version 27.0 (SPSS Inc., Chicago, IL, USA).

### 2.3 Study design

**Figure 1:**
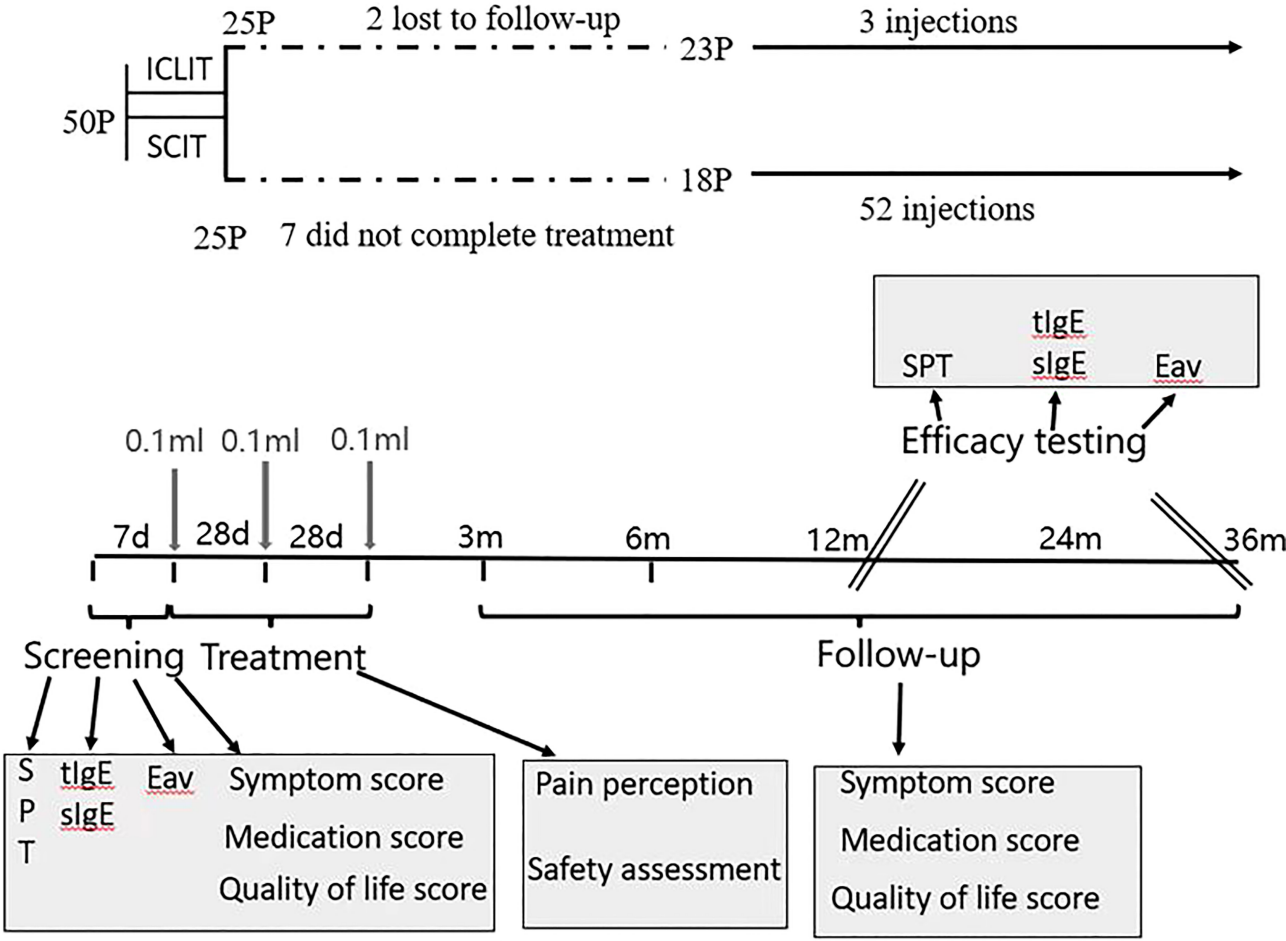
Study design. The trial included 1 pre-treatment screening visit, 3 treatment visits, 5 follow-up visits within 36 months after treatment. ICLIT, intra-cervical lymphatic immunotherapy; SCIT, subcutaneous immunotherapy Eav, eosinophils absolute value; SPT, skin prick test; tIgE, total IgE; sIgE, specific IgE.

This is a prospective randomized controlled study with 25 patients receiving ICLIT (**Figure 1**), another 25 patients received SCIT as the control group for the study.

Eligibility was determined at the first visit when patients were required for a questionnaire including allergic symptoms, use of rescue medication, and quality of life. Also, The qualification of patients was determined by SPT and collecting blood samples of patients for evaluating eosinophils absolute value, total serum dust mite IgE and Derp and Derf-specific IgE. Based on the results of the allergy tests, and in accordance with the principle of randomization, patients received the first injection of Arroger-standardized dust mite allergen extract (Novo- Helisen-Depot, Allergopharma GmbH &Co. KG, Reinbek, Germany) with the specific injection regimen shown in 2.4. Patients’ pain perception and adverse reactions that occurred need to be recorded throughout the treatment. Patients were followed-up by hospital visit or phone interview for a questionnaire related to the first visit at 3 month, 6 month,12 month, 24 month and 36 month after treatment. Besides, SPT and blood sample were required at 12-month and 36-month follow-up so as to assess the effectiveness of treatment.

### 2.4 Treatment process

#### 2.4.1 ICLIT group

Injections were performed under color Doppler ultrasound guidance using a 7-gauge needle, three times on the same side. Under strict aseptic procedures, 0.1ml (50TU) of 500TU/mL (concentration grade 2) of alum-adsorbed allergen extracts was injected into the superficial II or III area cervical lymph nodes (approximately 0.5-0.8 cm in size), after 28 days (4 weeks) and 56 days (8 weeks), the second and third injections were the same. Treatment interval was 4 weeks for a total period of 2 months(**Figure 1**) (12). A pullback was required before each injection in order to prevent the drug being injected into the blood vessel. Patient vital signs, peak expiratory flow, local and/or systemic adverse reactions at the injection site were monitored at the hospital for 1 hour after each injection. If adverse reactions were observed, corresponding therapeutic measures were given according to the grading criteria (14), and details of all subsequent reactions within the next 24 hours were required to record and report by patients or their families.

#### 2.4.2 SCIT group

The injection site was either lateral to the distal third of the upper arm or dorsal to the middle third of the forearm, and treatment consisted of two phases which were dose accumulation and dose maintenance.

Allergen injection with a starting dose of 50 TU/mL was injected once a week for up to 15 weeks. In the first 12 weeks, patients were injected with (1) 0.1mL, 0.2mL, 0.4mL, and 0.8mL of allergen at 50 TU/mL (concentration grade 1); (2) 0.1mL, 0.2mL, 0.4mL, and 0.8mL of allergen at 500 TU/mL (concentration grade 2); (3) 0.1mL, 0.2mL, 0.4mL, and 0.6mL of allergen at 5000 TU/mL (concentration grade 3). In 13, 14 and 15 week, patients were injected with 0.8mL, 1.0mL and 1.0mL of allergen at 5000 TU/mL (concentration grade 3), respectively. Dose maintenance phase started after 15 weeks, during which the allergen injection 1.0mL dose of 5000 TU/mL (concentration grade 3) was kept unchanged, and the interval between each injection was 4 weeks with the total treatment course of 3 years. Evaluations of SCIT were in the same way as ICLIT.

### 2.5 Observation indicators

#### 2.5.1 Primary indicators

Primary outcomes were assessed before treatment (baseline), at 3 month, 6 month, 12 month, 24 month and 36 month, including (1) change in allergic symptoms associated with dust mite was assessed by a four-scale system where 0=no symptoms, 1=mild symptoms, 2=moderate symptoms, and 3=most severe symptoms (15). At the same time, total nasal symptom scores (TNSS) and total ocular symptom scores (TOSS) were summed to obtain the total symptom score (TSS) ranging from 0 to 18; (2) rescue medication use associated with allergic symptoms was recorded as 1 point/day for nasal, ophthalmic and/or oral antihistamines, 2 points/day for nasal corticosteroids and 3 points/day for oral corticosteroids (15). Added the above score to obtain the total medication score (TMS) ranging from 0 to 6; (3) quality of life related to allergic symptoms was evaluated by rhinoconjunctivitis quality of life questionnaire (RQLQ), including 23 items in five domains: nasal symptoms, ocular symptoms, non-nasal/ocular symptoms, emotional functions, and behavioral functions (16). RQLQ was seven-point scale where 0=no distress and 6=extreme distress. Scores were summed to obtain the total quality of life score ranging from 0 to 150, and higher score indicated worse quality of life. All above scores were marked on the ruler with cartoon icon of facial expressions by patients themselves (or assisted by their families).

#### 2.5.2 Secondary indicators

Secondary outcomes included (1) pain perception during treatment was assessed by patients themselves (or assisted by their families) after each treatment using visual analogue scale (VAS). Scores were marked on the ruler with cartoon icon of facial expressions, ranging from 0 to 10 where 0=painless and 10=most painful. Total score was collected after the treatment (ICLIT ranged from 0 to 30 points and SCIT ranged from 0 to 520 points); (2) safety assessment was performed for each treatment by using a safety score table to record both local and systemic adverse reactions during treatment in all patients.

#### 2.5.3 Other secondary indicators

Other secondary outcomes were changes in circulating immunoglobulin level (serum dust mite-specific IgE) at baseline, 24 month and 36 month. Peripheral venous blood was taken and was separated by centrifugation at room temperature for 7 minutes at 3500 r/min, and then was stored at -80°C pending analysis. Serum Derp and Derf-specific IgE was detected using Thermo phadia 250 automatic fluorescence immunoassay analyzer and ImmunoCAP Specific IgE enzyme-labeled secondary antibody kit. Allergen criteria for detection were Derp and Derf positive with other allergens negative. The test results were graded from 0 to 6 according to Derp and Derf-specific IgE concentration. Grade 0 was defined as sIgE < 0.35 kU/L; Grade 1 was defined as sIgE ≥ 0.35 kU/L; Grade 2 was defined as sIgE ≥ 0.70 kU/L; Grade 3 was defined as sIgE ≥ 3.5 kU/L; Grade 4 was defined as sIgE ≥ 17.5 kU/L;Grade 5 was defined as sIgE ≥ 50 kU/L; Grade 6 was defined as sIgE ≥ 100 kU/L.Besides, Grade 0 indicated allergy (-) while Grade 1-6 indicated varying degrees of allergy,and Grade 1 and above was considered positive for sIgE.

### 2.6 Ethics and permissions

The institutional review board of the First People’s Hospital of Foshan approved this randomized controlled study (approval number [2018]-10) and all participants or their families signed informed consent. This study was registered in Chinese Clinical Trial Registry (clinical trial registration number: ChiCTR1800017130).

### 2.7 Statistical analysis

Normal distribution was decided by D’Agostino-Pearson omnibus normality test.Continuous variables were expressed as mean (standard deviation) and categorical variables were presented as n (%).Intragroup comparation was evaluated by paired t test or Wilcoxon Signed Rank test for normally distributed and non-normally distributed continuous variables,respectively. Numerical differences between two groups were assessed by unpaired t test or Mann-Whitney U test for normally distributed and non-normally distributed continuous variables, respectively.Changes in allergen species and SPT results were assessed by Fisher’s exact test or Pearson chi-square test. The threshold for significance was P<0.05. All statistical analyses were conducted using GraphPad Prism software, Version 9.0 (GraphPad Software, San Diego, California, USA).

## 3 Results

### 3.1 General information

**Figure 2:**
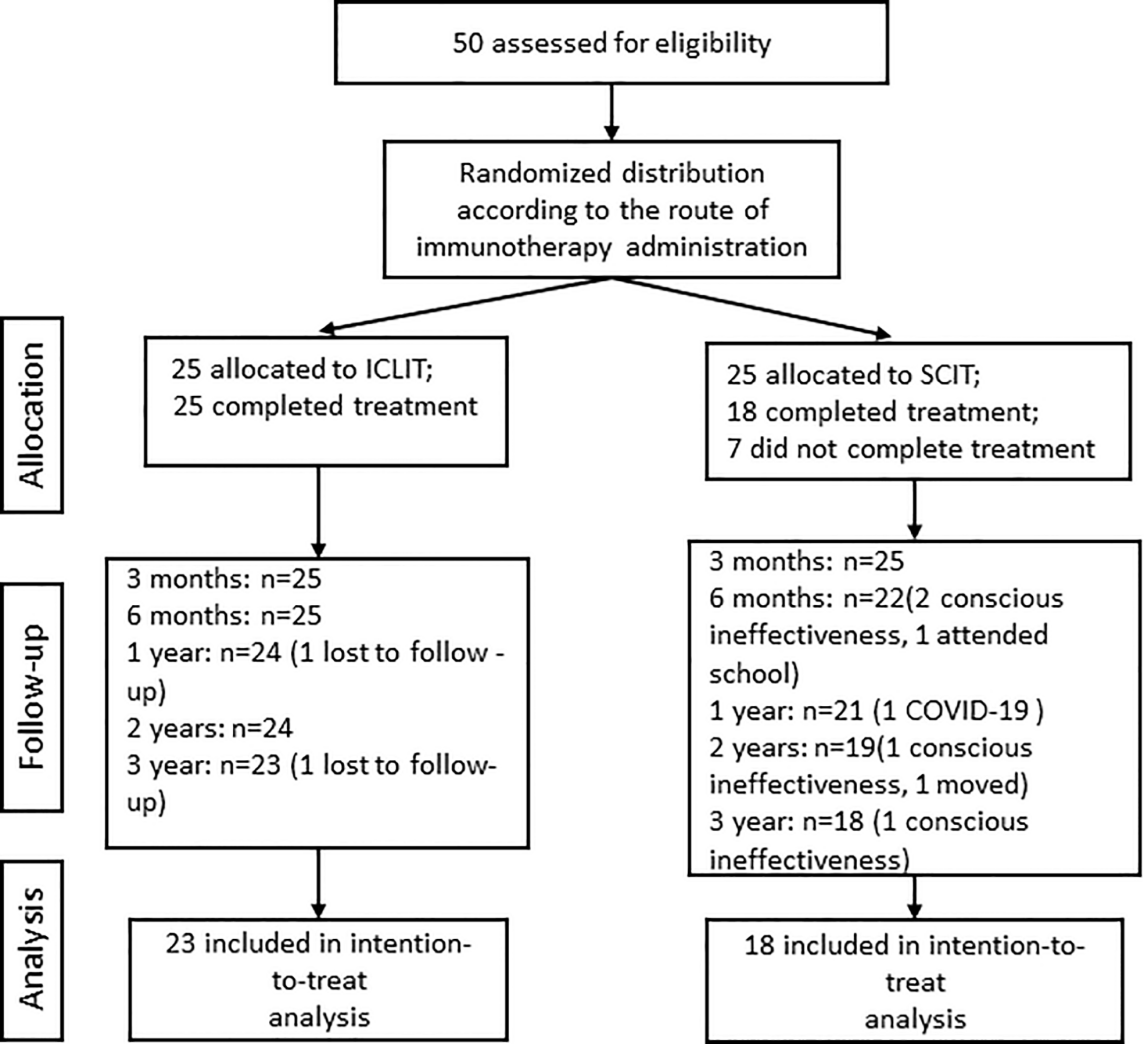
Research flow chart. ICLIT, intra-cervical lymphatic immunotherapy; SCIT, subcutaneous immunotherapy.

A total of 50 eligible patients were randomly divided into ICLIT group (n=25) and SCIT group (n=25) (**Figure 2**). All patients in ICLIT group completed the treatment while 7 patients in SCIT group did not complete the treatment, including 4 cases conscious ineffectiveness, 1 case could not visit hospital due to COVID-19, 1 case attended school, and 1 case moved. 2 patients were lost to follow-up in ICLIT group. 23 patients and 18 patients were finally included in ICLIT group and SCIT group, separately.

As shown in **Table 1**, there were no significant differences in baseline information between two groups, including age, gender, eosinophils absolute value, symptom score, and allergen-specific IgE, with all p values greater than 0.05. Also, patients in both two groups presented as Derp and Derf allergies with SPT results of +++ in most patients.

**Table 1:** Baseline characteristics by treatment group.

Characteristics	Treatment group		P-value
	ICLIT (n=23)	SCIT (n=18)	
Age, mean (SD),y	9.2 (2.8)	11.4 (3.5)	0.78
Gender (%)			0.54
male	13 (56.5)	12 (66.7)	
female	10 (43.5)	6 (33.3)	
Eosinophils absolute value, mean (SD),×10^9^/ L	0.5 (0.2)	0.4 (0.3)	0.21
Symptom score, mean (SD),P	12.3 (1.9)	12.8 (2.1)	0.52
Allergen(%)			1.00
Derp	0 (0.0)	0 (0.0)	
Derp +Derf	23 (100.0)	18 (100.0)	
Allergen-total IgE, mean (SD),KU/L	774.5 (796.0)	480.5 (397.1)	0.35
Allergen-specific IgE, mean (SD), KU/L			
Derp	66.2 (31.7)	71.3 (26.1)	0.19
Derf	71.6 (33.1)	61.4 (28.7)	0.58
SPT(%)			0.66
++	4 (17.4)	4 (22.2)	
+++	14 (60.9)	12 (66.7)	
++++	5 (21.7)	2 (11.1)	

SPT, skin prick test; SI, skin index; ++, 0.5≤SI<1;+++, 1≤SI<2;++++, SI≥2。 Analysis and comparison of two groups of data: continuous variable data in accordance with normal distribution adopts unpaired t test, and non normal distribution adopts Mann-Whitney U test; Fisher's exact test or Pearson chi-square test is used for categorical variable data.

### 3.2 Primary outcomes

#### 3.2.1 ICLIT had a significant long-term effect

**Figure 3:**
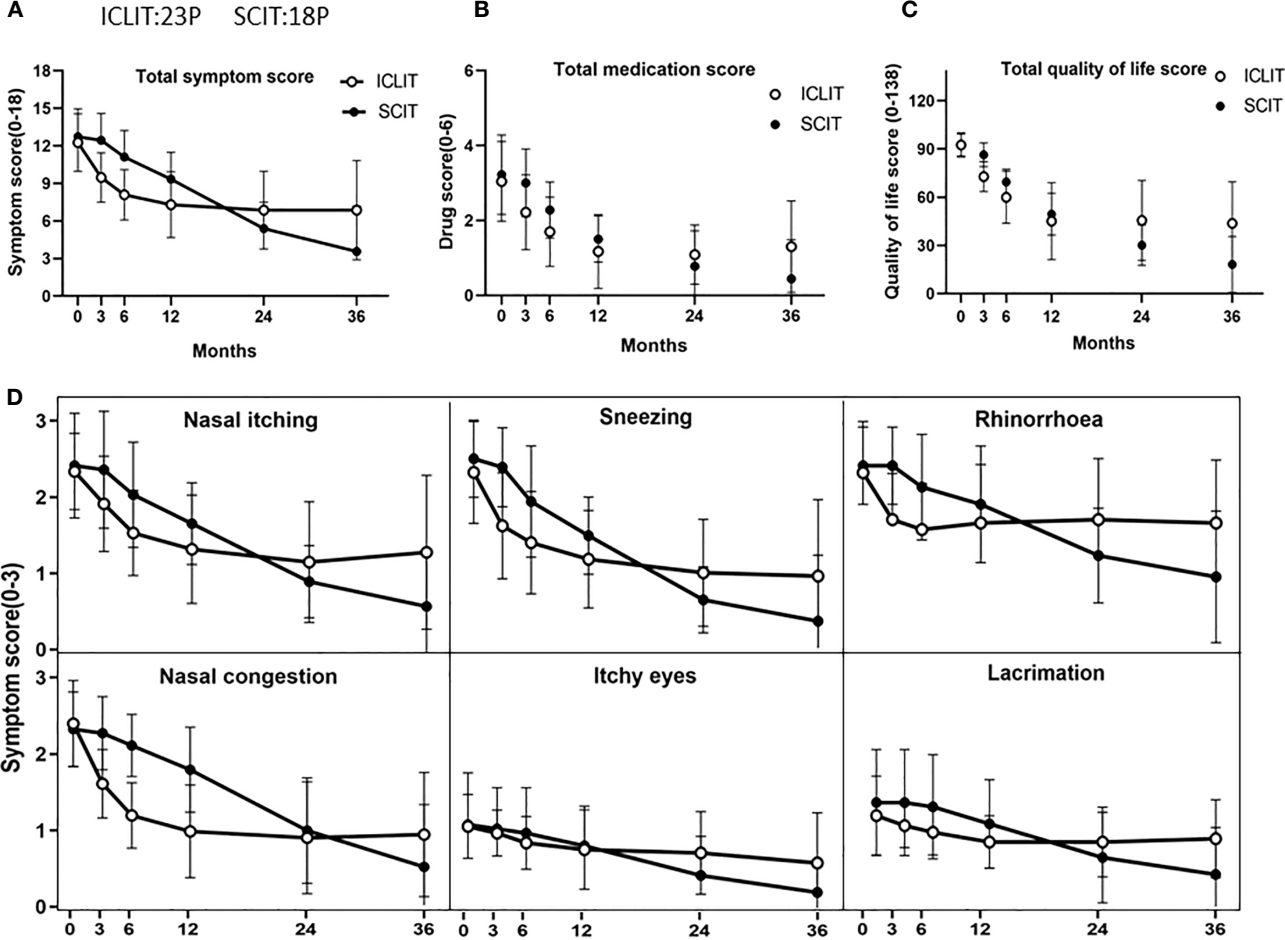
Comparison of overall efficacy between ICLIT and SCIT. Changes in TSS **(A)**, TMS **(B)**, total quality of life score **(C)**, TNSS and TOSS **(D)** of patients receiving ICLIT and SCIT before treatment, 3 months, 6 months, 12 months, 24 months and 36 months after treatment. ICLIT,intra-cervical lymphatic immunotherapy; SCIT, subcutaneous immunotherapy; TSS, total symptom scores; TMS, total medication scores; TNSS, total nasal symptom scores; TOSS, total ocular symptom scores.

At baseline in ICLIT group, TNSS was 10.3 (1.7), TOSS was 2.4 (0.8), TSS was 12.3 (1.9), TMS was 3.5 (1.3), and total quality of life score was 92.8 (4.9). At baseline in SCIT group,TNSS was 10.4 (1.3), TOSS was 2.6 (1.2), TSS was 12.8 (2.1), TMS was 3.1 (1.0), and total quality of life score was 92.3 (7.5). No significant differences were found between the two groups in the above scoring system (p > 0.05). At 3, 6, 12, 24, and 36 month of treatment, scores at each time point were shown as follows. TNSS in ICLIT group were 7.7 (1.6), 6.7 (1.7), 6.1 (2.2), 5.7 (2.6), and 5.8 (3.3). TOSS in ICLIT group were 2.1 (0.5), 2.0 (0.8), 1.7 (0.7), 1.7 (0.9), and 1.6 (1.0). TSS in ICLIT group were 9.4 (1.7), 8.4 (2.0), 7.9 (2.9), 7.5 (3.3), and 7.8 (4.1). TMS in ICLIT group were 2.7 (1.2), 2.3 (1.0), 1.7(1.1), 1.3 (0.9), and 1.7 (1.2).Total quality of life scores in ICLIT group were 73.3 (9.1), 61.4 (15.2), 45.5 (24.0), 47.2 (25.3), and 45.3 (26.3). TNSS in SCIT group were 10.2 (1.3), 9.0 (1.4), 7.5 (1.5), 4.3 (1.6), 3.0 (2.8). TOSS in SCIT group were 2.5 (1.1), 2.4 (1.1), 1.9 (0.8), 1.2 (0.9), 0.7 (1.0). TSS in SCIT group were 12.5 (2.0), 11.3 (2.0), 9.1 (1.9), 5.4 (2.2), 3.6 (3.7). TMS in SCIT group were 2.9 (0.9), 2.2 (0.7), 1.4 (0.6), 0.6 (0.8), 0.3 (0.8). Total quality of life scores in SCIT group were 86.9 (6.8), 69.3 (7.5), 48.6 (11.9), 30.4 (11.6), 17.6 (16.1). Compared with baseline, children in both groups showed significant decrease in TSS (**Figure 3A**), TMS (**Figure 3B**), total quality of life score (**Figure 3C**) and all items of TNSS and TOSS (**Figure 3D**) at 36 months with all p values less than 0.0001.

#### 3.2.2 ICLIT improved the overall efficacy more quickly

At 3 month of treatment, TSS in ICLIT group and SCIT group was 9.4 (1.7) and 12.5 (2.0), separately. Change of TSS in ICLIT group was 2.9 (1.2) with a decreased rate of 22.7% and change of TSS in SCIT group was 0.3 (0.5) with a decreased rate of 2.2%. ICLIT group showed significant degree of symptom improvement compared with SCIT group (p < 0.0001) (**Figure 3A**).

#### 3.2.3 ICLIT was not as effective as SCIT in a long term

**Figure 4:**
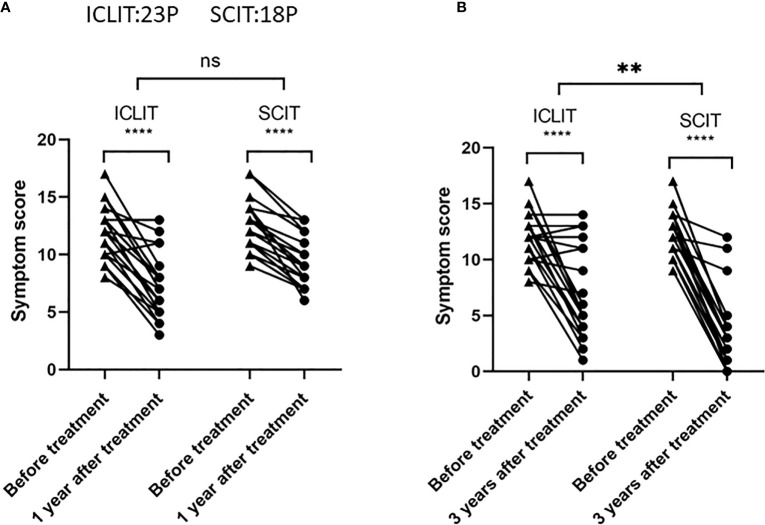
Comparison of short-term (1 year later) and long-term (3 years later) effects between ICLIT and SCIT. Compared with the two groups, **(A)** there is no difference in short-term effect; **(B)** There is significant difference in long-term effect. ns has no difference, * * p<0.01 ; **** p<0.0001. ICLIT,intra-cervical lymphatic immunotherapy; SCIT, subcutaneous immunotherapy.

Short-term efficacy and long-term efficacy were observed for allergic symptoms at the first and third year after treatment in both groups. At baseline, there was no significant difference in TSS between ICLIT group (12.3 (1.9)) and SCIT group (12.8 (2.1)). At 1 year after treatment, TSS in ICLIT and SCIT group was 7.9 (2.9) and 9.1 (1.9), separately, indicating the significant symptom improvement in both groups. At 3 year after treatment, TSS in ICLIT and SCIT group was 7.8 (4.1) and 3.6 (3.7), separately, indicating the significant symptom improvement in both groups. In summary, ICLIT (ICLIT=4.7, SD=2.9) and SCIT (SCIT=3.6, SD=1.9) shared similar short-term efficacy (**Figure 4A**), but ICLIT (ICLIT=4.8,SD=4.7) had poorer long-term efficacy than SCIT (SCIT=9.2,SD=3.8) (**Figure 4B**).

#### 3.2.4 ICLIT significantly improved the symptoms of most allergic rhinoconjunctivitis children

**Figure 5:**
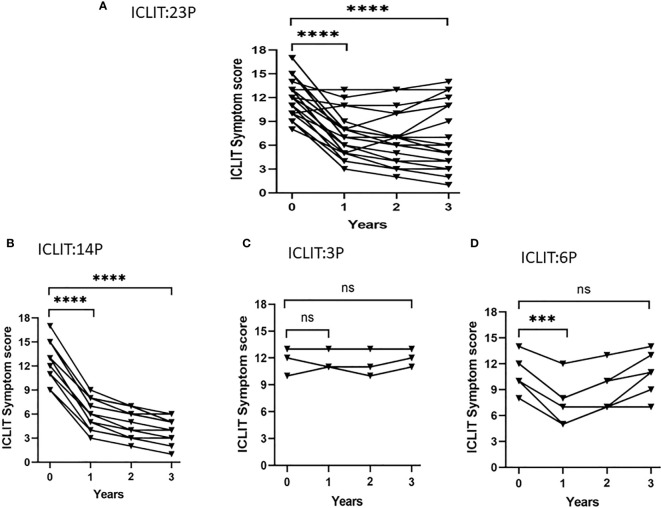
Symptom changes after ICLIT treatment. Compared with baseline, **(A)** 23 patients had significant symptom improvement (p < 0.0001) in both short-term (1 year after treatment) and long-term (3 year after treatment); **(B)** 14 patients with significant symptom improvement in both short-term and long-term after treatment; **(C)** 3 patients without significant symptom improvement in short-term or long-term after treatment; **(D)** 6 patients with significant symptom improvement in short-term, but poor symptom control in long-term. *** p<0.001 ; **** p<0.0001. ICLIT, intra-cervical lymphatic immunotherapy. ns represents p > 0.05.

Short-term efficacy and long-term efficacy were observed for allergic symptoms at the first and third year after treatment only in ICLIT group. After undergoing ICLIT, 23 patients had significant symptom improvement (p < 0.0001) in both short-term (1 year after treatment) and long-term (3 year after treatment) (**Figure 5A**), and were further divided into 3 clusters.Cluster 1 referred to symptom improvement, including 14 patients with significant symptom improvement in both short-term and long-term after treatment (**Figure 5B**). Cluster 2 referred to ineffectiveness, including 3 patients without significant symptom improvement in short-term or long-term after treatment (**Figure 5C**). Cluster 3 referred to poor efficacy, including 6 patients with significant symptom improvement in short-term, but poor symptom control in long-term (p = 0.70) (**Figure 5D**).

### 3.3 Secondary outcomes

#### 3.3.1 ICLIT had less pain

**Figure 6:**
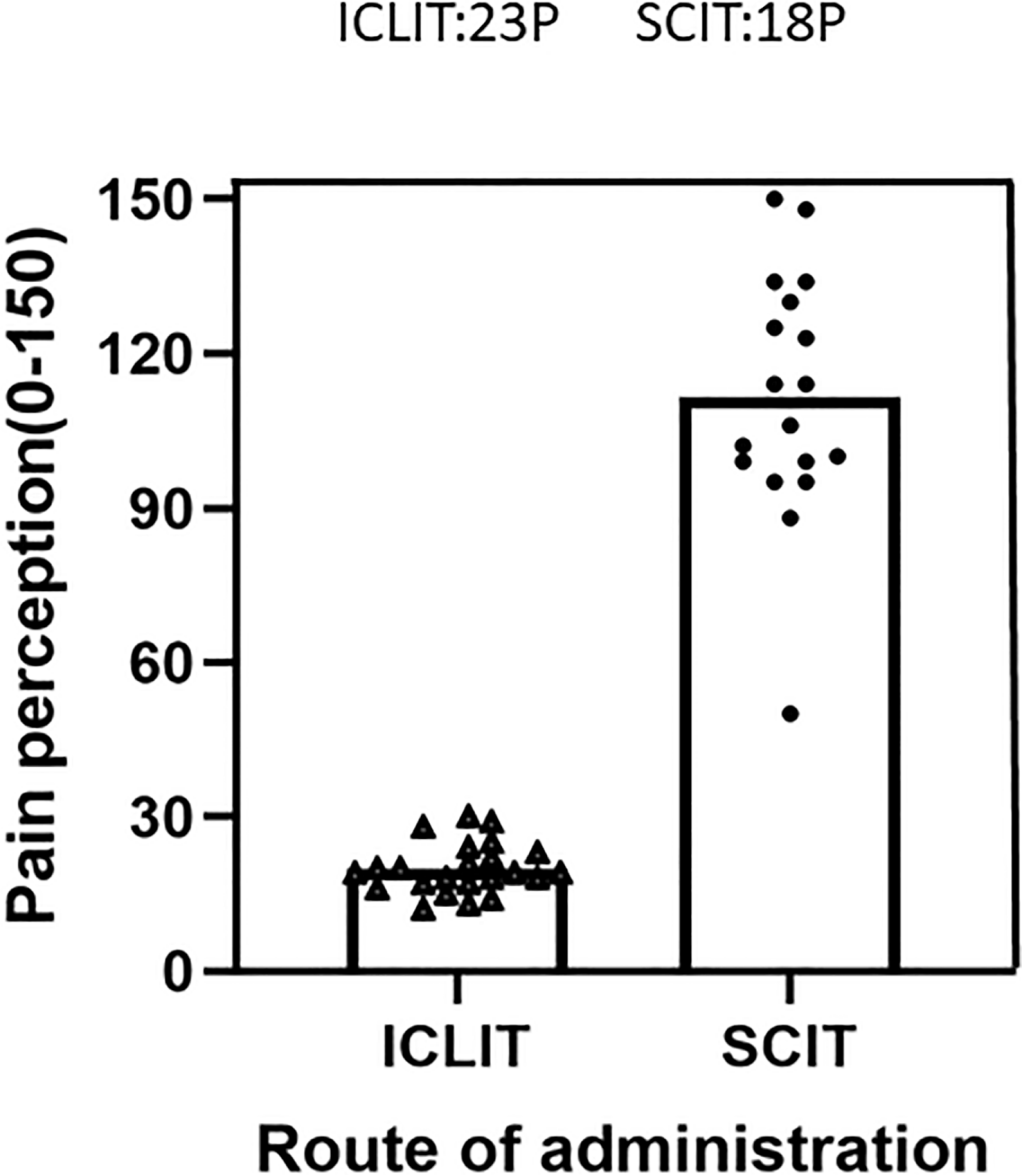
Pain perception were compared between patients in ICLIT and SCIT groups. The total pain score of patients injected into cervical lymph nodes and veins was recorded. ICLIT, intra-cervical lymphatic immunotherapy; SCIT, subcutaneous immunotherapy.

**Figure 6** displayed pain perception of the two groups after completing the treatment. The total VAS score was 19.56 (4.90) after 3 times of ICLIT and was 111.44 (24.18) after 52 times of SCIT. Children in ICLIT group felt significantly less pain than those in SCIT group.

#### 3.3.2 ICLIT had higher safety

**Table 2** presented adverse reactions during the treatment in both groups. 23 patients in ICLIT group received a total of 69 injections, the adverse events were all mild local adverse reactions (1 lymph node swelling/irritation, 1 itching around the puncture site, and 1 redness around the puncture site). All adverse reactions disappeared within 24 hours without use of rescue medications. No moderate to severe local adverse reactions or systemic adverse reactions were observed. 18 patients in SCIT group received a total of 936 injections and 152 adverse events were reported, including 13 systemic mild to moderate adverse reactions and 1 severe adverse reaction. 0.15 mL and 0.3 mL of epinephrine solution (1:1000) were used immediately for closed injection around the allergen injection site, and blood pressure and pulse were continuously monitored. All adverse events subsided within 48 hours and the following treatment was performed as planned.

**Table 2:** Adverse events associated with intra-cervical lymphatic and subcutaneous injections.

	ICLIT (69 injections)		SCIT (936 injections)		P-value
	Number of injections		Number of injections		
Local reactions(%)					
Lymph node swelling/ Irritation	Mild: Moderate: Severe:	1 (1.4) 0 (0.0) 0 (0.0)	Mild: Moderate: Severe:	31 (3.3) 12 (1.3) 3 (0.3)	
Itch	Mild: Moderate: Severe:	1 (1.4) 0 (0.0) 0 (0.0)	Mild: Moderate: Severe:	30 (3.2) 11 (1.2) 1 (0.1)	
Redness	Mild: Moderate: Severe:	1 (1.4) 0 (0.0) 0 (0.0)	Mild: Moderate: Severe:	38 (4.1) 10 (1.1) 2 (0.2)	
Systemic reactions(%)					
Nasal symptoms	Mild: Moderate: Severe:	0 (0.0) 0 (0.0) 0 (0.0)	Mild: Moderate: Severe:	5 (0.5) 2 (0.2) 1 (0.1)	
Urticaria and angioedema	Mild: Moderate: Severe:	0 (0.0) 0 (0.0) 0 (0.0)	Mild: Moderate: Severe:	4 (0.4) 1 (0.1) 0 (0.0)	
Gastrointestinal infection	Mild: Moderate: Severe:	0 (0.0) 0 (0.0) 0 (0.0)	Mild: Moderate: Severe:	1 (0.1) 0 (0.0) 0 (0.0)	
Total		3 (4.3)		152 (16.2)	0.0053

Local adverse reactions mainly include redness, itching, induration, rash, etc. at the injection site. Mild local adverse reactions refer to a diameter of ≤ 4 cm, moderate local adverse reactions refer to a diameter of>4 cm and>15 minutes, both of which disappear within 24 hours. Severe local adverse reactions with a diameter of>4 cm can lead to pseudopodia, which can occur immediately or within 15 minutes; Systemic adverse reactions are divided into 5 levels: Level 1: no systemic reactions (asymptomatic), Level 2: mild systemic reactions (rhinitis, local urticaria, and mild asthma), Level 3: moderate systemic reactions (slow onset, systemic urticaria, and/or moderate asthma), Level 4: severe systemic reactions (rapid onset, systemic urticaria, muscular vascular edema, and/or severe asthma) Grade 5: anaphylactic shock (rapid onset of systemic urticaria, flushing, itching, asthma attack, wheezing, hypotension, etc.). The data of the two groups are consistent with categorical variable, and Fisher's exact test is used for data analysis and comparison.

### 3.4 Other secondary outcomes

#### 3.4.1 ICLIT significantly decreased serum dust mite-specific IgE levels

**Figure 7:**
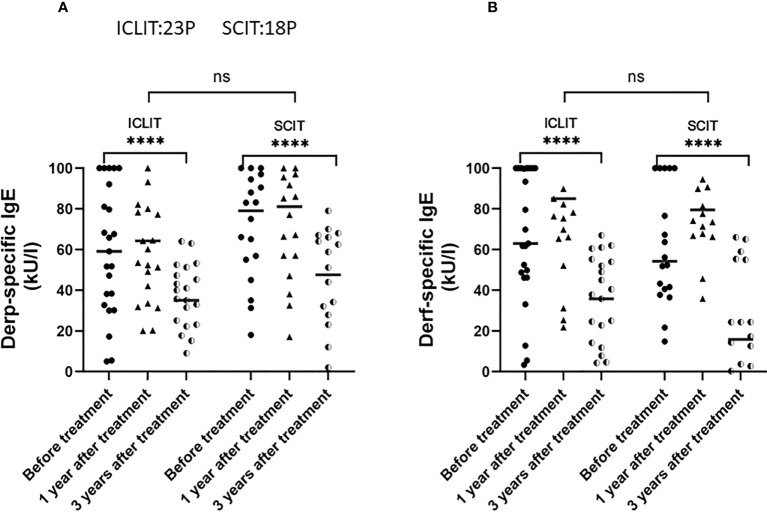
Changes of serum Derp sIgE **(A)** and Derf sIgE **(B)** in patients receiving ICLIT and SCIT at baseline, 1 year and 3 years after treatment. sIgE, specific IgE **** p<0.0001. ns represents p > 0.05.

sIgE was an important pathogenic antibody of AR, there were no significant differences in serum Derp and Derf sIgE between the two groups at baseline. After 3 years of treatment, serum Derp and Derf sIgE levels significantly decreased in both groups, but no significant difference in the changes was found between the two groups (**Figures 7A, B**).

## 4 Discussion

In recent years, pediatric allergic rhinoconjunctivitis has become a global health issue and one of the most concerned allergic diseases. As the first-line treatment recommended by Allergic Rhinitis and its Impact on Asthma (ARIA) guideline and European Academy of Allergy and Clinical Immunology (EAACI) guideline (17, 18), AIT has been confirmed to prevent the deterioration of symptoms caused by allergens and the serious impact caused by complications (19, 20). During the pandemic of COVID-19, pediatric patients and their families had less outdoor activities and hospital visits. The ideal AIT should achieve good clinical efficacy with a small dose in a short period of time and can mediate long-term immune tolerance, so the optimal plan is to shorten the overall time of treatment and improve the benefit of treatment (21).

In 2008, Senti et al. (22) first conducted a randomized double-blind controlled clinical trial of intralymphatic immunotherapy (ILIT). A total of 165 pollen allergic patients were randomly divided into a 2-month course ILIT group (n=58) or 3-year course SCIT group (n=54), and results showed that efficacy of 2-month ILIT was equivalent to that of 3-year SCIT. In 2013, Hylander et al. (23) treated grass/birch-induced allergic rhinoconjunctivitis patients with ILIT in an open-label pilot study and a double-blind controlled trial and proved that allergic symptoms decreased during the pollen season after both ILIT and SCIT treatments, while no significant improvement in symptoms was observed in the placebo control group. In addition to evaluating the clinical efficacy of ILIT in patients with grass pollen allergy, Lee (24) and Park (25) also evaluated the efficacy of ILIT in patients with Derp and Derf, cat, dog, or mixtures thereof, in which Lee (24) noted a rapid improvement in quality of life and allergy symptoms in patients with allergy to those indoor inhalant allergens after ILIT, whereas Park (25) noted that patients’ overall symptom scores and nasal reactivity to dust mite allergens after ILIT were not significantly different from the control group in a randomized double-blind placebo-controlled trial, which clearly contradicts previous studies, so the article mentioned that more studies are needed in the future to elucidate the clinical effectiveness of ILIT. certainly, most studies have shown that ILIT has significant therapeutic effects.Although clinical efficacy and safety of ILIT among allergic rhinoconjunctivitis patients have been verified by several clinical trials, the mechanism of immune tolerance of allergic rhinoconjunctivitis is more closely related to cervical lymph nodes (26). Besides, several important organs in th inguinal region can lead to inconvenience in performing ultrasound-guided puncture, which further involves exposing patient privacy. In 2018, our team proposed a new immunotherapy for allergic rhinoconjunctivitis through three allergen injections into cervical lymph nodes instead of inguinal lymph nodes, and confirmed that ICLIT treatment had significant short-term efficacy and high safety among allergic rhinoconjunctivitis patients (12). Since there remained a lack of ICLIT studies in terms of long-term efficacy and its comparison with the traditional SCIT, this study conducted a prospective randomized study with the SCIT as controls. Our results pointed out all patients in ICLIT group completed the treatment while 7 patients withdrew in SCIT group. By interviewing with patients’ families, withdrawal reasons included complicated treatment procedure and conscious ineffectiveness. Compared with the conventional immunotherapy, ICLIT served as a rapid and effective novel immunotherapy which was more attracted to children and their families, and was more receptive and selective.

In this study, we observed significant decreases in TNSS and TOSS, less dependence of rescue drugs, and improvements of quality of life at 3, 6 and 12 month after treatments. Also,at 24 and 36 month after treatments, patients in ICLIT and SCIT maintained the improvement of symptoms and quality of life, indicating that ICLIT had sustained and stable efficacy as SCIT.

After 1 month of treatments, allergic rhinoconjunctivitis patients in ICLIT group had better improvements than SCIT group in terms of allergic symptoms, dependence on rescue drugs and quality of life, indicating that ICLIT could rapidly mediate immune tolerance. An animal experiment injected an equivalent amount of allergen into one area and found that the dose injected into the lymph nodes was approximately 100-fold higher than that injected subcutaneously into the vein (27). Another study reported that direct injection of allergen into lymph nodes significantly increased the stimulation of specific immune responses and antigen expression (28). Thus, for children with moderate to severe allergic rhinoconjunctivitis, ICLIT can rapidly alleviate allergic reactions, decrease the occurrence of complications such as asthma, and reduce the impact of allergic diseases on the physical and mental health.

We compared both short-term and long-term efficacy between ICLIT group and SCIT group.It was found that the two groups had comparable 1-year short-term efficacy and consistent production of immune tolerance. Besides, ICLIT group had poorer 3-year long-term efficacy than SCIT group. After detailed investigation, patients in ICLIT group could be divided into 3 clusters. Cluster 1 referred to symptom improvement, including 14 patients with significant symptom improvement in both short-term and long-term after treatment. Cluster 2 referred to ineffectiveness, including 3 patients without significant symptom improvement in short-term or long-term after treatment. Also, they had no irritation response to ICLIT throughout the treatment period. Considering that these patients were insensitive to immunotherapy, molecular mechanisms could be investigated in this group of children in the future. Cluster 3 referred to poor efficacy, including 6 patients with significant symptom improvement in short-term, but poor symptom control in long-term. The larger proportion of Cluster 3 resulted in the lower long-term symptom control in ICLIT group than that in SCIT group. After analysis of symptom improvement, we found that they actually had a higher sensitivity to ICLIT treatment at the beginning but gradually decreasing sensitivity over the long term, which was similar with the traditional immunotherapy. Tseng et al. (29) and Frew et al. (30) explored the poor long-term efficacy of traditional immunotherapy and suggested that the reasons might be too low maintenance dose or insufficient course of treatment. Regarding dose adjustment, the correlation between clinical efficacy and clinical efficacy has been studied many times in ILIT. Scholars such as Park (25) and Hellkvist (31) proposed that dose escalation has no significant effect on patients with allergic rhinitis through randomized double-blind placebo-controlled trials, and even excessive dose can cause allergic reactions in patients, and cannot further improve their quality of life and nasal mucosal irritation response.However, in 2012, Senti et al. (32) conducted an ILIT randomized double-blind controlled trial, dividing 20 patients with rhinoconjunctivitis allergic to cat dander into two groups, and received three doses (dose Gradually increasing) MAT-Feld-1 (recombinant cat antigen) after injection, no adverse reactions occurred. Compared with the control group (placebo), the nasal tolerance increased by 74 times after 3 injections of MAT-Feld-1, the specific IgG4 level in the serum increased by 5.66 times after 300 days, and the production of IL-10 was positively correlated with the IgG4 response It can significantly induce immune tolerance, and the quality of life and allergic symptoms of patients are significantly improved. In 2016, Patterson et al. (33) conducted a double-blind parallel randomized controlled study on 15 patients with rhinoconjunctivitis allergic to grass pollen. Seven patients in the treatment group received 3 dose-increasing doses and preseason superficial inguinal lymph node injections (0.1, 0.2 and 0.5 ml), none of the patients had serious adverse reactions, the symptoms were significantly improved after ILIT, and the drug usage was significantly reduced. This study first verified that it is safe for patients under 18 years old to use increasing doses of ILIT. It is a tolerable intra-lymph node immunotherapy regimen. However, no relevant research has been conducted in ICLIT. Then, for the poor long-term curative effect in Cluster3, whether the long-term curative effect and immune tolerance of the patients can be improved by adjusting the dose, so that the patients under the same number of allergen injections, children can achieve clinical benefits comparable to traditional immunotherapy, which requires further verification. Regarding the course of treatment, in 2019, Konradsen et al. (34) conducted a randomized double-blind placebo-controlled trial in patients with rhinoconjunctivitis allergic to grass pollen, by investigating 3 times of ILIT before the season and injecting a booster dose one year later (1000 SQ-U) ILIT, confirmed that ILIT after booster injection is safe, effectively improved the patient’s allergic symptoms and reduced the use of drugs. In 2021, Weinfeld et al. (35) conducted a double-blind placebo-controlled randomized trial through ILIT preseason booster treatment in allergic rhinitis patients allergic to grass pollen. The study confirmed that booster can improve allergen specificity compared with placebo increased IgG4 levels, improved allergic reactions caused during grass pollen season, and had fewer side effects. The above studies have confirmed that the booster injection can benefit patients from the booster injection after 1 year of treatment. Then, for patients with high sensitivity to allergens in ICLIT at the beginning, but the sensitivity decreases in the long run, the 1-year timely injection of booster may increase the sensitivity of children to allergen preparations, improve their allergic symptoms for a long time, and at the same time regulate the level of serum immunoglobulin, essentially inducing immune tolerance. Therefore, if the dose and frequency of allergen injections can be optimized, ICLIT may become an attractive treatment option in allergic rhinoconjunctivitis in the future.

Safety remains a major concern during immunotherapy among pediatric patients with allergic rhinoconjunctivitis. Severe local adverse reactions and systemic adverse reactions may cause laryngeal edema, resulting in partial or complete obstruction of the airway, leading to life-threatening adverse events such as hoarseness, dysphonia, and even dyspnea. Therefore, before treatment, we should carry out emergency plans, the main emergency equipment includes: lathes that can lie flat, bedside breathing, pulse, blood pressure and blood oxygen saturation monitors, syringes, needles, intravenous infusion pumps and other items used to open venous channels, oxygen tanks, suction tubes, stethoscopes, laryngoscopes, tracheal tubes, masks, respiratory air bags, dental pads and other intubation items and instruments to relieve airway obstruction; The main emergency drugs include: antihistamines, rapid-acting β2 agonists, epinephrine solution (1:1000), and other emergency drugs. In this study, 23 patients with ICLIT underwent a total of 69 injections, and only 3 mild local adverse reactions occurred, 1 lymph node swelling/irritation, 1 itching around the puncture site, and 1 redness around the puncture site.All of them relieved within 24 hours without using emergency equipment or medication. There were no moderate to severe local adverse events or systemic adverse events during treatment. This trial is the same as ILIT causing only minor adverse effects in most previously reported studies (22, 23, 32, 33), but several studies have reported serious local adverse effects (36, 37) and systemic adverse effects (37–39) in ILIT, which, of course, are related to factors such as the initial dose of the allergen and allergen preparation; A total of 936 injections were performed in 18 cases of SCIT, and in addition to varying degrees of local adverse reactions, 14 systemic adverse events occurred, and emergency drug epinephrine solution (1:1000) was used for first aid, and emergency equipment was not used.Compared with SCIT, ICLIT showed superior safety,which might be related to the fact that the allergen dose required for a single injection into lymph nodes was approximately 1/100 of that required for subcutaneous injection. In addition, in the preparation of allergens, Lee et al. (24) and Park et al. (25) used aqueous allergen extracts and L-tyrosine-adsorbed allergen extracts in ILIT, respectively, and found that both of them can cause severe allergic reactions by applying them to patients with allergic rhinitis, so in this article, as in most studies, we used alum-adsorbed allergen extracts, which could achieve a long-lasting effect and therefore prolong the release time.Furthermore, we observed the much less pain in ICLIT group than SCIT group, which was consistent with Senti et al. (22) who proposed that the sensory nerve distribution in lymph nodes was sparse and pain from intralymphatic injection was limited to skin. ICLIT not only had a significant long-term effect, but greatly increased safety and reduced pain response, which was specifically beneficial for pediatric allergic rhinoconjunctivitis patients and their families.

The principle of immunotherapy is to reduce the pathogenic antibody IgE, especially the serum specific IgE antibody, and to increase the protective antibody IgG at the same time. Studies have shown that specific IgE competes with IgG4 antibody for binding allergens, and successful SCIT refers to an increase in serum specific IgE at the beginning and a slow decrease for a long time period. In this study, we found that levels of Derp IgE and Derf IgE decreased after treatment of ICLIT and SCIT, and the degree of reduction was similar between the two treatments.

There are some limitations in this study. First, this study is open-label study, so patients’ and investigators’ expectation to ICLIT and SCIT (placebo effect) and spontaneous improvement of natural course might affect on the study. Additionally,this was a single-center prospective randomized controlled study with a relatively small sample size. Second, we only investigated changes in IgE levels before and after treatment and did not include the protective antibody IgG4. Therefore, multicenter study with larger sample size is required in the future, as well as that for the molecular mechanisms of ICLIT-induced immune tolerance. ICLIT may become a new option among all AIT treatments.

## 5 Conclusion

This study first demonstrated that ICLIT could significantly relieve the allergic symptoms, improve the safety and compliance, and reduce the pain response among children with allergic rhinoconjunctivitis. In summary, ICLIT was a novel immunotherapy that could improve the clinical symptoms, save time and economic costs of children with allergic rhinoconjunctivitis.

## Data availability statement

The original contributions presented in the study are included in the article/supplementary material. Further inquiries can be directed to the corresponding authors.

## Ethics statement

The studies involving human participants were reviewed and approved by the institutional review board of the First People’s Hospital of Foshan. Written informed consent to participate in this study was provided by the participants’ legal guardian/next of kin.

## Author contributions

QW wrote this manuscript and data analysis, KW independently completed the research design and concept, YQ collected data and followed up patients, YX and YG processed statistical data on the manuscript, WH, YL, and QY revised and proofread the manuscript, RZ reviewed and provided important guidance on the manuscript, JT solved complex issues in the manuscript, and made final review and finalization. All authors listed have made a substantial, direct, and intellectual contribution to the work and approved it for publication.
